# HIF1α‐mediated AIMP3 suppression delays stem cell aging via the induction of autophagy

**DOI:** 10.1111/acel.12909

**Published:** 2019-01-31

**Authors:** Chul Kim, Ji‐Min Park, Youngsook Song, Sunghoon Kim, Jisook Moon

**Affiliations:** ^1^ Department of Biotechnology, College of Life Science CHA University Pocheon-si Korea; ^2^ Medicinal Bioconvergence Research Center Seoul National University Seoul Korea; ^3^ Department of Molecular Medicine and Biopharmaceutical Sciences, Graduate School of Convergence Technology Seoul National University Suwon Korea

**Keywords:** aging, AIMP3, autophagy, HIF1α, Notch3, stem cells

## Abstract

Senescence in stem cells, which occurs as a consequence of chronic responses to the environment, defines the capacity of stem cells for proliferation and differentiation as well as their potential for tissue regeneration and homeostasis maintenance. Although stem cells reside under low oxygen pressure and the availability of oxygen is known to be a crucial determinant in their fate, the key modulators in stem cell aging and the underlying mechanism have yet to be unraveled. Human placenta‐derived mesenchymal stem cells (hpMSCs) were cultured under hypoxia (3% O_2_) or normoxia (21% O_2_) to investigate the key factors that regulate stem cell senescence under hypoxic conditions. RNA sequencing results suggested that the expression of aminoacyl‐tRNA synthetase‐interacting multifunctional protein 3 (AIMP3, EEF1E1), an aging inducer, in the hpMSCs was dramatically repressed under hypoxia with concurrent suppression of the aging marker p16^INK4a^. The hpMSCs that overexpressed AIMP3 under hypoxic conditions displayed significantly decreased proliferation and fewer stem cell characteristics, whereas the downregulation of AIMP3 ameliorated the age‐related senescence of MSCs. Consistent with the results of the hpMSCs, MSCs isolated from the adipose tissue of AIMP3‐overexpressing mice exhibited decreased stem cell functions. Interestingly, AIMP3‐induced senescence is negatively regulated by hypoxia‐inducible factor 1α (HIF1α) and positively regulated by Notch3. Furthermore, we showed that AIMP3 enhanced mitochondrial respiration and suppressed autophagic activity, indicating that the AIMP3‐associated modulation of metabolism and autophagy is a key mechanism in the senescence of stem cells and further suggesting a novel target for interventions against aging.

## 1 INTRODUCTION

Cellular senescence represents an irreversible cell cycle arrest and is considered one of the main causes of aging in an individual (Falandry, Bonnefoy, Freyer, & Gilson, 2014). Several stressors and signals have been suggested to cause aging, including reactive oxygen species (ROS)‐associated protein and DNA damage, proteasomal and lysosomal dysfunction, altered extracellular signaling, increased inflammatory responses, replicative telomere attrition, and endoplasmic reticulum and mitochondrial stresses. The accumulating stressors converge onto cell cycle arrest mediated by p16^INK4a^, p19^ARF^, or p53. The aged cells then affect inflammation and the aging of an individual by secreting specific combinations of growth factors, cytokines, and chemokines called the senescence‐associated secretion phenotype (SASP; Lopez‐Otin, Blasco, Partridge, Serrano, & Kroemer, 2013; Rayess, Wang, & Srivatsan, 2012; Rodier & Campisi, 2011). Given that the disruption of homeostasis induced by stem cell senescence is one of the fundamental causes of increased cellular senescence and that stem cell aging is a major risk factor for human diseases at various ages, such as cancer, neurodegeneration, weakness and metabolic diseases (Oh, Lee, & Wagers, 2014; Signer & Morrison, 2013), understanding the mechanism of stem cell aging is a prerequisite for preventing cell senescence and further clinical approaches.

The microenvironment around stem cells under low oxygen pressure, called a niche, is suitable for promoting self‐renewal and proliferation via the induction of pluripotent genes, including Oct‐4 and Klf‐4 (Park et al., 2013). Stem cells exposed to hypoxia display increased viability, prolonged proliferation, and differentiation capacities along with delayed aging processes, including the inhibition of telomere shortening during replication and the suppression of HIF1α‐TWIST‐mediated E2A‐p21 (Buravkova, Andreeva, Gogvadze, & Zhivotovsky, 2014; Tsai et al., 2011). Hypoxia‐inducible factor (HIF), a nuclear heterodimeric transcription factor, is known as a key regulator in responses to hypoxia and consists of one of three α subunits (1α, 2α, and 3α) and one of two β subunits (ARNT and ARNT2). The HIF complex regulates the expression of genes containing the hypoxia response element (HRE: ACGTG) that controls stress adaptation, angiogenesis, glucose metabolism, proliferation, survival, and senescence in stem cells as well as in somatic cells (Brahimi‐Horn & Pouyssegur, 2009; Nakayama, 2009).

Considering the fact that hypoxic conditions are able to impede stem cell senescence, we investigated the mechanism by which HIF1α inhibits stem cell senescence and explored a novel regulatory pathway in stem cell aging using human placenta amnion‐derived mesenchymal stem cells (hpMSCs). Compared with the cells cultured under normal oxygen conditions, 21% O_2_, the hpMSCs under hypoxic conditions, 3% O_2_, remained proliferative and survived for longer periods with the inhibition of p16^INK4a^ and p53. RNA sequence analysis revealed that aminoacyl‐tRNA synthetase‐interacting multifunctional protein 3 (AIMP3), a key aging‐inducing factor, was suppressed under hypoxia. This result was confirmed by in vitro assays: both the hpMSCs expressing AIMP3 and the adipose‐derived stem cells from AIMP3‐overexpressing mice, AIMP3 TG, exhibited accelerated stem cell aging and dysfunction. We found that the AIMP3 level in hpMSCs was negatively regulated by HIF1α and positively regulated by Notch3 and that the combination of HIF and Notch signals determined the cellular behaviors of hpMSCs. In addition, HIF1α mediated the repression of AIMP3‐induced autophagy and suppressed mitochondrial respiration, whereas the overexpression of AIMP3 compromised autophagy function in the adipose stem cells from AIMP3 TG mice. In this study, for the first time, we found that AIMP3 is a key molecule in the hypoxia–autophagy‐associated antiaging pathway and that the AIMP3‐autophagy axis is a new therapeutic target for aging.

## 2 RESULTS

### 2.1 The hpMSCs cultured under hypoxia displayed a prolonged proliferation capacity and maintained their characteristics with passages

**Figure 1:**
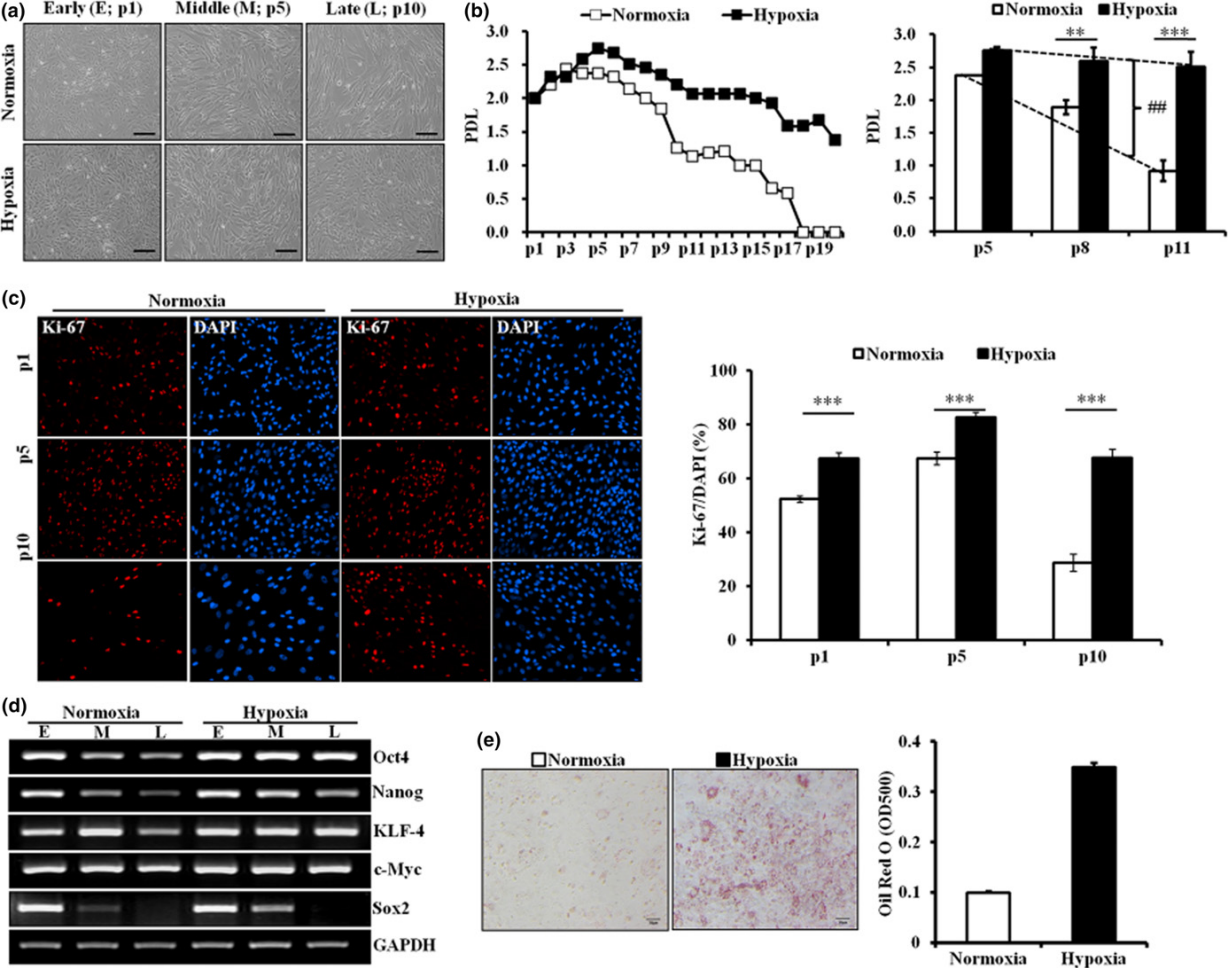
Hypoxic conditions prolonged the proliferative capacity and the maintenance of the stemness of hpMSCs. (a) The isolated hpMSCs were cultured long‐term under normoxia (21% O_2_) or hypoxia (3% O_2_), and their morphological changes were observed. The cells under normoxia became larger, whereas the hpMSCs under hypoxic conditions maintained their shape up to the late passage. Early: passage 1 (p1), Middle: p5, and Late: p10. The scale bars represent 100 μm. (b) Population doubling level (PDL) of hpMSCs under normoxia declined from p5, and the cells stalled growth at approximately p15–18. However, the hpMSCs under hypoxia maintained their ability to proliferate up to p15. The PDL of the hpMSCs under normoxia was also significantly reduced at a given passage and trend over passages compared to that under hypoxia. **p ≤ 0.01; ***p ≤ 0.001: ^##^ *p* ≤ 0.01. (c) The Ki‐67 staining of different passages displayed a clear reduction of proliferative cells in normoxia at p1, 5, and 10. ***p ≤ 0.001. (d) The hpMSCs under hypoxia maintained the expression of the stem cell‐specific transcription factors Oct4, Klf4, and c‐Myc over p10, but their expression under normoxia was suppressed from p5. The expression of other markers, Nanog and Sox2, declined in both conditions but the decline was much faster in normoxia. (e) Oil Red O staining confirmed the hypoxia‐enhanced adipogenic differentiation. The hpMSCs cultured under hypoxia preferentially differentiated into adipocytes. The scale bars represent 50 μm

To investigate the responses of hpMSCs to low oxygen pressure, cells were separately maintained either under hypoxia, 3% O_2_, or normoxia, 21% O_2_, immediately after isolation, which was considered passage 0 (p0). No significant difference was observed in the cellular morphology between the two groups up to p5. However, while large and flattened heterogeneous hpMSCs, a senescent phenotype, were frequently found at under normoxia at later passages, p10, the hpMSCs under hypoxia maintained their homogenous morphology at p10 and even later passages (Figure 1a). Reflecting a remarkable increase in the accumulated cells under hypoxia (Supporting Information Figure S1A), the population doubling level (PDL) of the hpMSCs under hypoxia was sustained up to p13‐p15, while the cells under normoxia became less proliferative from approximately p5 and then ceased growth by approximately p10 (Figure 1b). This prolonged proliferation under hypoxia was supported by significantly higher numbers of Ki‐67‐positive cells across all passages of cells grown under hypoxia than those grown under normoxia (Figure 1c). Although the expression of stem cell‐specific transcription factors, including Oct4, Nanog, KLF‐4, c‐Myc, and Sox2, declined with passages under normoxia (Figure 1d), the oxygen availability did not affect their MSC characteristics along passages. Under both conditions, the hpMSCs at late passages, up to p10, were negative for embryonic (SEEA4, TRA‐1‐60, and TRA‐1‐81) and hematopoietic stem cell markers (CD34) and positive for MSC markers (CD9 and CD44; Supporting Information Figure S1B). In addition, differential oxygen pressures influenced the differentiation potentials of hpMSCs (Figure 1e, Supporting Information Figure S1C).

### 2.2 The hypoxic culture condition suppresses cell death and senescence‐inducing pathways in hpMSCs at late passage

Given that the reduced potential for self‐renewal represents stem cell aging (Signer & Morrison, 2013), the enhanced proliferative character under hypoxia indicates that low oxygen pressure suppresses stem cell aging. We performed RNA sequencing (R‐seq) analysis of transcripts from the hpMSCs at p10 under normoxia and hypoxia to investigate which factors lead to the antiaging effect under hypoxia. Gene ontological analysis showed that 57 genes induced under hypoxia were strongly associated with the positive regulation of transcription and proliferation, aging, the MAPK cascade, the regulation of PI3K signals, and responses to estradiol, drug, and insulin, indicating that the hpMSCs respond to hypoxia via responses to external signals and the active modulation of gene expression and protein synthesis (Supporting Information Table S1). However, 18 genes significantly repressed under hypoxia were associated with apoptosis, the response to cellular stress such as UV, DNA damage, and unfolded protein response, and receptor tyrosine signaling, including Erb‐B2 receptor tyrosine kinase (ERBB2; Table 1), indicating that the prolonged capacity for self‐renewal under hypoxia is through the induction of proliferation and macromolecular synthesis and the inhibition of cellular stresses and cell death. Among the highly suppressed apoptosis‐related genes, the ARS interacting multifunctional protein 3 (AIMP3/EEF1E1) was notable because AIMP3‐overexpressing mice (AIMP3 TG), a model of progeria, manifest reduced lifespan, premature aging, reduced bone density, and wrinkled skin with reduced subcutaneous adipocytes (Oh et al., 2010), although it is unknown what role AIMP3 plays in stem cell senescence in response to differential oxygen pressures.

**Table 1:** The expression of multiple aging‐related genes was downregulated by oxygen pressure

GO enrichment of downregulated aging‐associated genes	Genes
Positive regulation of apoptotic process	*UBB, TNF, FAS,* EEF1E1*, BAK1*
ERBB2 signaling pathway	*UBB, HSP90AA1, PIK3R1*
Extrinsic apoptotic signaling pathway via death domain receptors	*TNF, FAS, PIK3R1*
Cellular response to UV	*PIK3R1, ATR, BAK1*
Intrinsic apoptotic signaling pathway in response to DNA damage	*TNF, PIK3R1, BAK1*
Response to glucocorticoid	*GHR, TNF, FAS*
Response to drug	*HSP90AA1, ATR, GCLM, BAK1*
Positive regulation of endoplasmic reticulum unfolded protein response	*PIK3R1, BAK1*
Activation of MAPK activity	*GHR, UBB, TNF*
Necroptotic signaling pathway	*TNF, FAS*

### 2.3 The stem cells derived from AIMP3‐overexpressing mice exhibited compromised stem cell properties

**Figure 2:**
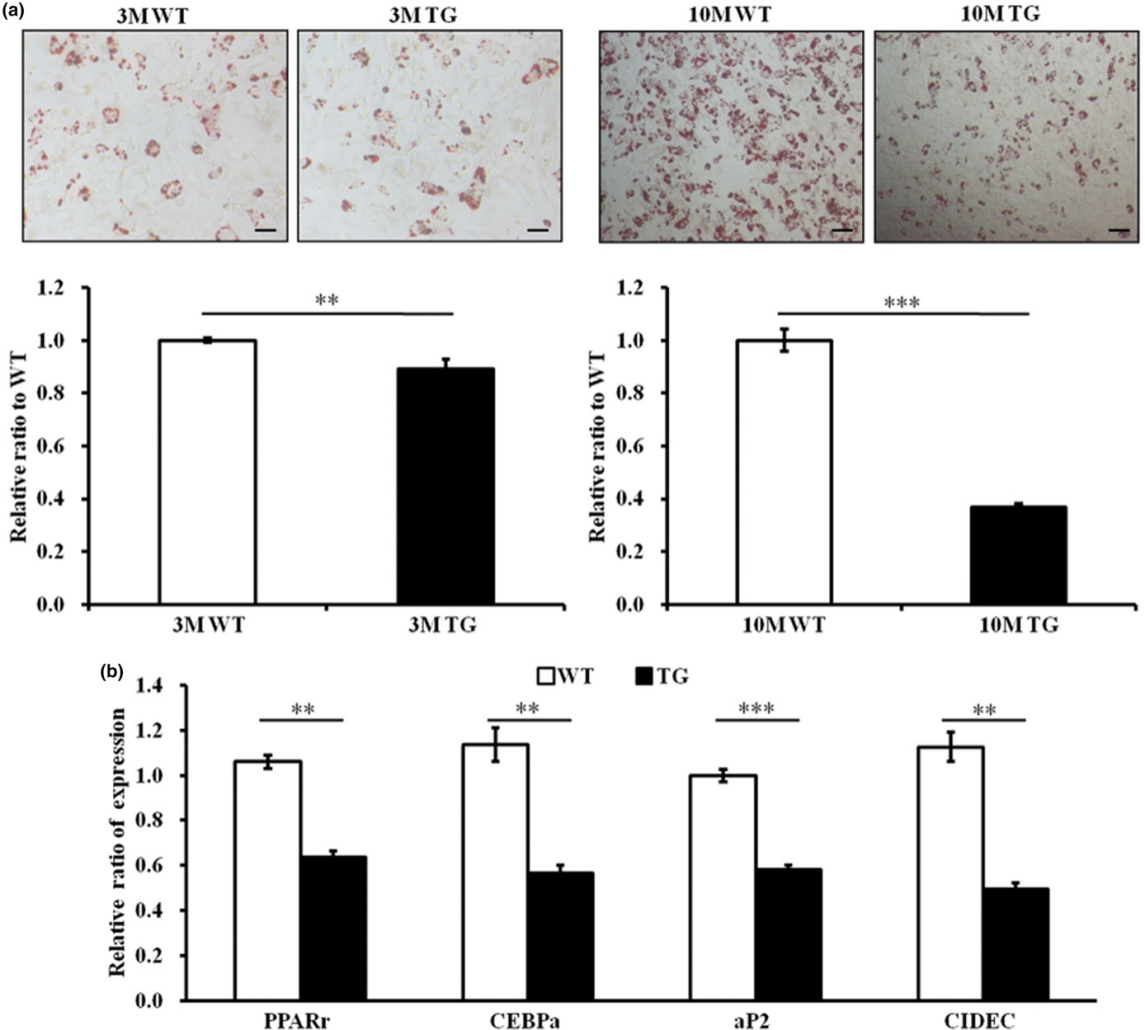
The stem cells derived from AIMP3 TG mice exhibited compromised stem cell properties. (a) Oil Red O staining with primary adipose‐derived MSCs (AD‐MSCs) from 3‐ and 10‐month‐old AIMP3 TGs (3 M and 10 M each) revealed compromised adipogenic potential in even 3 M AD‐MSCs compared to the littermate controls, and the differentiation deficits became more evident in the AD‐MSCs from 10 M AIMP3 mice. The scale bars represent 50 μm. WT *n* = 3 and AIMP3 TG *n* = 4 mice of each age. (b) Four key transcription factors for adipogenesis were significantly suppressed in AD‐MSCs from 3 M AIMP3 TG mice at p6. **p ≤ 0.01; ***p ≤ 0.001. Ap2: adipocyte fatty acid‐binding protein; CEBPα: CCAAT/enhancer‐binding protein alpha; CIDEC: cell death‐inducing DFFA‐like effector C; PPARγ: peroxisome proliferator‐activated receptors gamma. The 36B4 gene, an acidic ribosomal phosphoprotein P0 (RPLP0), was used for normalization

It is possible that the weakened skin elasticity in AIMP3 TGs results from the accelerated senescence‐induced differentiation deficits of the adipose‐derived MSCs (AD‐MSCs). To test this hypothesis, AD‐MSCs were isolated from 3‐ (3 M) and 10‐month‐old (10 M) AIMP3 TG female mice and were differentiated into adipocytes under normoxia because hypoxia is reported to suppress adipogenic differentiation in our system (Yun, Maecker, Johnson, & Giaccia, 2002). The reduced expression of stem cell factors such as Sox2, c‐Myc, and KLF4 was observed with increased levels of p57, a p53‐dependent senescence marker, in AIMP3 AD‐MSCs even from 3 M AIMP3 TG mice (Supporting Information Figure S2). Compared to that of the cells from age‐matched littermate controls, the AD‐MSCs from 3 M AIMP3 TG mice exhibited a significant reduction (approximately 13%) in differentiation. The extent of the compromised adipogenesis became more prominent with age: AIMP3 AD‐MSCs from 10 M TGs demonstrated approximately a 60% reduction in adipogenesis compared to the age‐matched controls (Figure 2a). Consistent with the Oil Red O staining results, the AD‐MSCs from 3 M TG mice displayed a significant reduction of adipogenic markers compared to controls (Figure 2b). This suggests that the forced expression of AIMP3 accelerates the senescence of AD‐MSCs and compromises their differentiation potentials with age.

### 2.4 Hypoxia inhibited AIMP3 expression and AIMP3‐associated stem cell senescence

**Figure 3:**
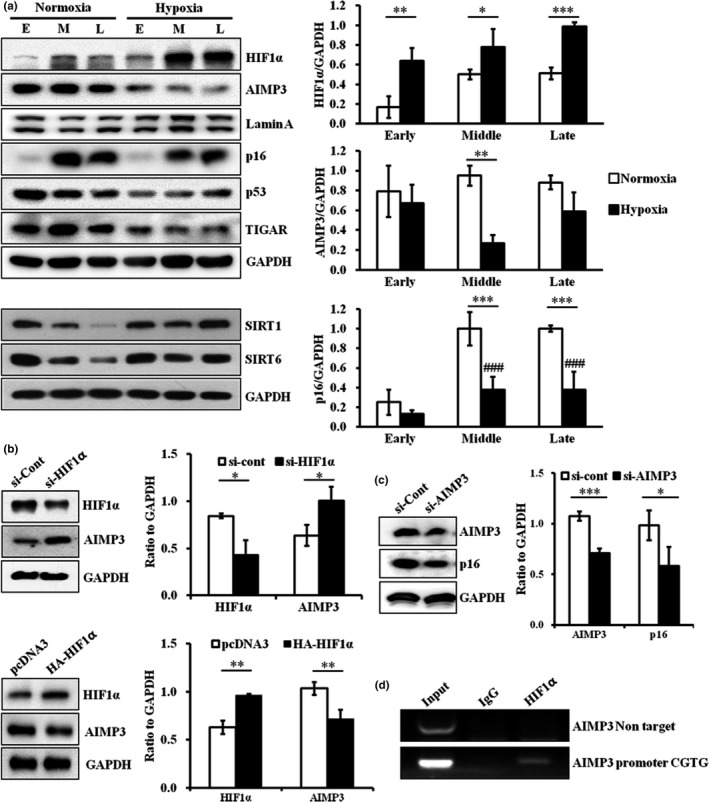
Hypoxia negatively regulated AIMP3 expression in a HIF1α‐dependent manner. (a) Hypoxia‐induced the expression of HIF1α, SIRT1, and SIRT6 and suppressed the expression of AIMP3 and p16^INK4a^, p53, and TIGAR. *p ≤ 0.05; **p ≤ 0.01; ***p ≤ 0.001. (b) The suppression of HIF1α induced AIMP3 under hypoxia at p5, whereas HIF1α overexpression under normoxia significantly repressed AIMP3. (c) Under normoxia, the reduction of AIMP3 suppressed p16^INK4a^ in the cells at p5, and the AIMP3‐overexpressing cells induced p16^INK4a^ under hypoxia. *p ≤ 0.05; ***p ≤ 0.001. (d) The ChiP assay with anti‐HIF1α showed that the AIMP3 promoter contains a binding site for HIF1α

Here, we suggest that AIMP3 transcription declines under hypoxia and that its forced expression accelerates stem cell aging. To investigate how AIMP3 expression is regulated and how it influences stem cell aging at low oxygen levels, hpMSCs were cultured under either normoxia or hypoxia and then were compared at different passages. The induction of HIF1α was observed under hypoxia, whereas the level of AIMP3 under hypoxia was significantly reduced compared to normoxia (Figure 3a). Along with AIMP3 reduction, the hypoxic condition suppressed the senescence factors p16^INK4a^ and p53 and induced the anti‐senescent sirtuins SIRT1 and SIRT6 in the hpMSCs across passages. In addition, the reduced TIGAR (TP53‐induced glycolysis and apoptosis regulator), an inhibitor of glycolysis and ROS‐mediated cell death, indicates that the hpMSCs under hypoxia rely less on mitochondrial respiration and are exposed to lower levels of stress (Bensaad et al., 2006; Wanka, Steinbach, & Rieger, 2012). The negative correlation between HIF1α and AIMP3 under hypoxia suggested negative regulation of AIMP3 by HIF1α. This hypothesis was supported by experiments using HIF1α RNA interference (siHIF1α) and overexpression: HIF1α suppression increased AIMP3 protein levels in the hpMSCs under hypoxia, whereas HIF1α overexpression under normoxia significantly reduced the AIMP3 protein levels (Figure 3b). As expected, the level of p16^INK4a^ was dependent on AIMP3 (Figure 3c). Because HIF1α regulates the expression of its target genes via binding to hypoxia response elements (HREs: G/ACGTG), we examined the HREs within 1,000 base pair from the AIMP3 transcriptional start site (Majmundar, Wong, & Simon, 2010). We found an incomplete putative HRE sequence at the −604 position from the start codon (Supporting Information Figure S3). Additionally, under hypoxia, the interaction between endogenous HIF1α and the putative HRE within the AIMP3 promoter region was confirmed by chromatin immunoprecipitation (ChIP) assay (Figure 3d), indicating that HIF1α is directly able to regulate AIMP3. Taken together, our results suggest the possibility that HIF1α negatively regulates aging, inducing AIMP3 via its binding to the AIMP3 promoter region and inhibiting stem cell aging under hypoxia.

### 2.5 AIMP3 expression was negatively modulated by HIF1α and Hey‐1 but positively modulated by Notch3

**Figure 4:**
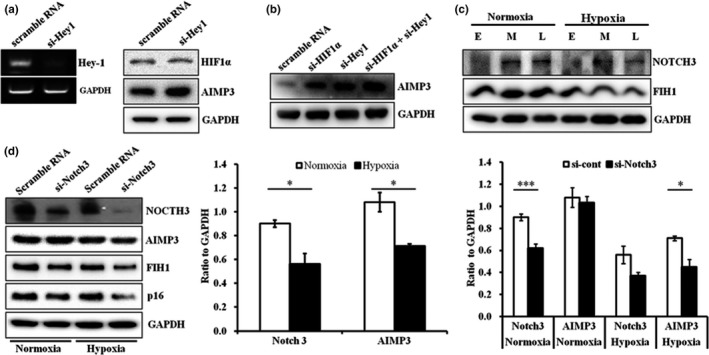
AIMP3 expression under hypoxia was suppressed by HIF1α‐Hey1 but induced by Notch3. (a) The suppression of Hey‐1 increased AIMP3 levels without influencing HIF1α expression. (b) The suppression of HIF1α and Hey‐1 additively elevated AIMP3 expression. (c) Notch3 expression was suppressed in the hpMSCs under hypoxia over passages with a concomitant reduction in FIH1. (d) The suppression Notch3 expression downregulated AIMP3, FIH1, and p16^INK4a^ in the hpMSCs under hypoxia. AIMP3 in the hpMSCs under normoxia was not affected by Notch3 reduction. *p ≤ 0.05; ***p ≤ 0.001

Stem cells reside in specialized hypoxic microenvironments called niches, where the cooperation of HIF1α/HIF2α and Notch signals determines the self‐renewal, pluripotency, metabolism, and aging of stem cells (Mohyeldin, Garzon‐Muvdi, & Quinones‐Hinojosa, 2010). Among Notch signal components, Hey1 is reported to support stem cell maintenance under hypoxia, suggesting that both HIF1 and Hey1 are able to regulate stem cell aging (Gustafsson et al., 2005). To test whether HIF1 and Hey1 collaborate on the regulation of AIMP3 under hypoxia, we modulated the level of either Hey1 or HIF1α in the hpMSCs under hypoxia. Consistent with its roles in stem cells, suppression of Hey‐1 under hypoxia induced AIMP3 expression without altering the HIF1α level (Figure 4a). AIMP3 was induced in both si‐HIF1α‐ and si‐Hey1‐treated hpMSCs, and the synergistic accumulation of AIMP3 was observed in both si‐HIF1α‐ and si‐Hey1‐treated cells, indicating that both HIF1α and Hey1 negatively regulate AIMP3 in an additive manner (Figure 4b). Because Notch3, one of the Notch receptors, suppresses the proliferation of trophoblasts in the placenta and functions as a tumor suppressor by inducing cellular senescence (Cui, Kong, Xu, & Zhang, 2013; Zhao, Zhuang, Huang, Feng, & Lin, 2015), the differential expression of Notch3 was first examined under different oxygen levels. Under hypoxic conditions, the levels of Notch3 at the middle and late passages were repressed compared to those under normoxia (Figure 4c), and the forced suppression of Notch3 led to the significant repression of AIMP3 and decreased p16^INK4a^ (Figure 4d), indicating that Notch3 is able to induce senescence via AIMP3 upregulation. A HIF1α inhibitor, factor inhibiting HIF1 (FIH1), which is known to modulate Notch3 signaling under different oxygen levels, exhibited results parallel to Notch3: FIH1 was suppressed under hypoxia and in si‐Notch3‐treated cells (Zheng et al., 2008). Contrary to its role, the reduced levels of Notch3 under normoxia did not lead to AIMP3 suppression, suggesting that Notch3 suppression is not sufficient to downregulate AIMP3 under normoxia. Taken together, these results suggest that two aging inducers, AIMP3 and p16^INK4a^, under hypoxia were suppressed by HIF1α and Hey‐1 in a synergistic manner and were induced by Notch3.

### 2.6 AIMP3 inhibited the autophagy‐associated antiaging mechanism

**Figure 5:**
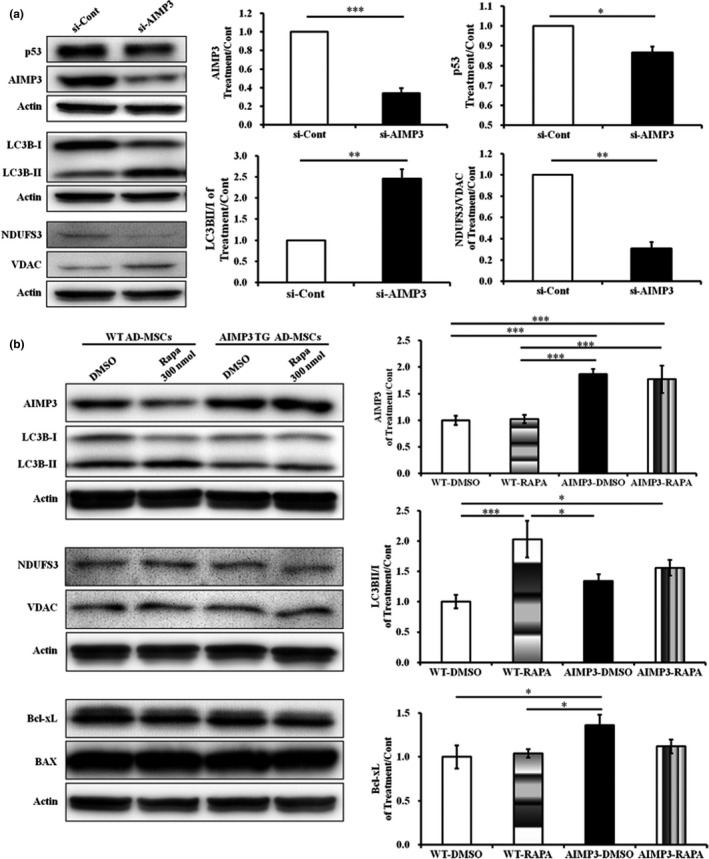
AIMP3 modulates mitochondrial biogenesis and autophagy‐associated stem cell aging. (a) Under normoxia, the hpMSCs treated with siAIMP3 displayed a significant increase in the LC3BII/I ratio compared to that of the control cells, indicating autophagy induction. In addition, siAIMP3‐treated hpMSCs showed a remarkable significant reduction in the NDUFS3/VDAC ratio as well as p53, suggesting the repression of mitochondrial respiration in AIMP3‐suppressed cells. *p ≤ 0.05; **p ≤ 0.01; ***p ≤ 0.001. (b) AD‐MSCs from 3‐month‐old AIMP3 TG mice exhibited compromised responses to autophagy‐inducing rapamycin. The rapamycin‐treated wild‐type AD‐MSCs showed significant induction of the LC3BII/I ratio, whereas AIMP3 TG AD‐MSCs were not responsive, indicating compromised autophagy activation. In addition, AIMP3 AD‐MSCs maintained a significantly increased level of Bcl‐xL. The level of AIMP3 was not altered in either WT or AIMP3 TG AD‐MSCs. *p ≤ 0.05; ***p ≤ 0.001

The suppression of AIMP3 was concomitant with alterations in apoptotic and metabolic regulators such as p53, TIGAR, SIRT1, and SIRT6 (Figure 3a), and a protein sequence similarity analysis showed that AIMP3 is similar to NDUFS3, a subunit of the mitochondrial respiratory chain complex I (RC‐CI; GeneCards database https://genecards.weizmann.ac.il). We then speculated whether AIMP3 regulates the metabolic status of stem cells as well as cell death under different oxygen pressures. The suppression of AIMP3 (siAIMP3) under normoxia resulted in a significant decrease in the ratio of NDUFS3 to voltage‐dependent anion channel (VDAC), a representative mitochondrial protein, with a reduction in p53 implying that AIMP3 positively regulates mitochondrial respiration (Figure 5a). Because autophagy is inversely related to mitochondrial respiration in stem cell aging, an autophagy marker, light chain 3B (LC3B), was examined to establish whether AIMP3 influences autophagy (Guan et al., 2013). In the si‐AIMP3‐treated hpMSCs, a significant increase in the levels of both an active form of LC3B, LC3BII, and the ratio of LC3BII/I was observed, indicating that the reduction of AIMP3 leads to autophagy activation in the cells. To investigate the AIMP3‐associated modulation of autophagy in endogenous stem cells, AD‐MSCs from 3‐month‐old AIMP3 TG mice were isolated and treated with an autophagy inducer, rapamycin (300 nmol), under normoxia for 24 hr. Compared to the wild‐type (WT) AD‐MSCs, AIMP3 AD‐MSCs (AIMP3‐DMSO) had almost twofold (1.87) increased expression of AIMP3, and the AIMP3 levels in both WT‐RAPA and AIMP3‐RAPA were not affected by rapamycin (Figure 5b). The rapamycin treatment significantly increased both LC3BII levels (1.46‐fold) and the ratio of LC3BII/I (twofold) in WT‐RAPA. The basal level of LC3BII in AIMP3 AD‐MSCs (AIMP3‐DMSO) was comparable to that in WT‐DMSO but AIMP3 AD‐MSCs were not responsive to rapamycin (AIMP3‐RAPA): although the amount of LC3BII and the ratio of LC3BII/I in AIMP3‐RAPA were significantly higher than those in WT‐DMSO and lower than those in WT‐RAPA, they were comparable to those in AIMP3‐DMSO. These results indicate that increased AIMP3 suppressed autophagy activation in endogenous stem cells. Neither AIMP3 nor rapamycin influenced the ratio of NDUFS3/VDAC in endogenous AD‐MSCs, in contrast to hpMSCs. Interestingly, one of the antiapoptotic proteins, Bcl‐xL, was significantly increased in AIMP3‐DMSO compared to that of both WT‐DMSO and WT‐RAPA, and rapamycin treatment suppressed Bcl‐xL in AIMP3 AD‐MSCs to a comparable level of that in WT‐DMSO and WT–RAPA, suggesting that rapamycin is able to modulate AIMP3‐associated cell survival signals. Taken together, the results demonstrated that the level of AIMP3 in hpMSCs and endogenous AD‐MSCs is inversely correlated with autophagy activation and influences mitochondrial function, including respiration and apoptosis.

## 3 DISCUSSION

Although the underlying mechanism is still elusive, many lines of evidence suggest that adverse conditions such as increased oxidative stress cause the aging‐associated stem cell dysfunction and depletion that lead to the reduction in tissue homeostasis and regeneration capacity in the aged (Lopez‐Otin et al., 2013). Assuming that stem cell deterioration leads to the physiological disturbance of tissue maintenance that occurs with age, it is very important to understand the aging‐related changes in the factors affecting stem cell aging and to find ways to prevent stem cell senescence. To understand the influence of the environment, particularly oxygen levels, on stem cell aging, we cultured hpMSCs either under normoxia or hypoxia and investigated the oxygen‐associated aging mechanisms and aging‐inducing factors. Consistent with previous reports showing delayed growth arrest of cytotrophoblasts in the placenta under hypoxic conditions (Genbacev, Zhou, Ludlow, & Fisher, 1997), the hpMSCs cultured under hypoxia maintained stem cell characteristics, including self‐renewal capacity and surface markers, for a longer period and their proliferation under normoxia declined after several passages (Figure 1). The stem cell dysfunctions in the hpMSCs under normoxia in this study are partly due to replication‐ and oxidative stress‐induced cellular aging as evidenced by the following: (a) the upregulation of p53 and TIGAR, a glycolysis inhibitor, under normoxia indicates that the cells rely more on mitochondrial respiration at the expense of ROS generation and cellular damage (Moussavi‐Harami, Duwayri, Martin, Moussavi‐Harami, & Buckwalter, 2004); (b) the expression of p16^INK4a^ increased and the levels of SIRT1 and SIRT6 declined under normoxic conditions compared with those of hypoxic conditions (Figure 3a); (c) genes promoting proliferation and mitosis were suppressed, and apoptotic genes were induced under normoxia compared to the gene expression under hypoxia (Table 1 and Supporting Information Table S1); and (d) HIF1α and the bHLH protein Hey‐1 both negatively regulated an aging‐inducing AIMP3‐p16^INK4a^ axis in hpMSCs under hypoxia (Figure 4) as evidenced by ability of the HIF1α–TWIST axis (TWIST is another bHLH protein) to induce proliferation and to repress the ROS‐induced senescence in the bone marrow‐derived MSCs cultured through many passages under hypoxia (Tsai, Yew, Yang, Huang, & Hung, 2012).

AIMP3/p18 is a member of a macromolecular protein complex consisting of several different aminoacyl‐tRNA synthetases (ARSs; the multi‐tRNA synthetase complex, MSC) and regulates protein synthesis and multicellular responses including immune response, proliferation, and death (Kim, Hur, Kim, Yoo, & Lee, 2011; Kwon et al., 2011). Human AIMP3 is known as a potent tumor suppressor via the induction of ATM/ATR‐p53‐mediated cell cycle arrest in response to DNA damage, and the systemic depletion or haploidy of AIMP3 was reported to cause massive DNA damage in human cancers. In response to DNA damage, AIMP3 can dissociate from the MSC and translocate to the nucleus for DNA repair and induce ATM/ATR‐p53‐mediated cell cycle arrest (Park et al., 2006). Recently, the induced depletion of AIMP3 was reported to cause severe DNA damage and to show a phenocopy of acute radiation syndrome in adult mice (Kim, Kim et al., 2018), leading to embryonic stem cell death with increased DNA damage (Kim, Jeon, Kim, & Jang, 2018). All of these findings support the functional significance of AIMP3 for DNA integrity. On the other hand, increased levels of AIMP3 are observed in aged human tissues and cells, and mice overexpressing AIMP3 (AIMP3 TGs) displayed accelerated aging processes via interactions with lamin A. These phenotypes could be reversed by microRNAs targeting AIMP3, miR‐543, and miR‐590‐3‐p (Lee et al., 2014; Oh et al., 2010). Both *Drosophila* and human AIMP3 contain a putative glutathione transferase domain that is capable of making protein–protein interactions as well as modulating cellular metabolism and metabolism‐induced cellular fates (flybase.org; Kim et al., 2008). These results suggest that AIMP3 is a key determinant for controlling aging, tumorigenesis, and stemness; thus, its optimal level in the cell should be tightly regulated to prevent aberrant cell fate determination.

**Figure 6:**
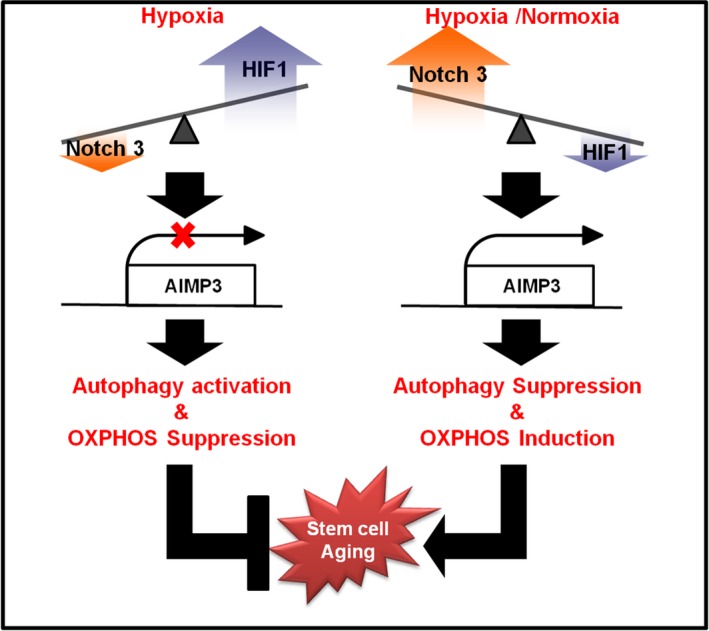
AIMP3 is a key modulator in autophagy‐associated antiaging mechanisms in stem cells. In stem cells under hypoxia, HIF1 is able to bind to a promoter region and to suppress the expression of AIMP3 in an additive manner with Hey‐1. The stem cells with repressed AIMP3 are able to activate autophagy and to reduce mitochondrial OXPHOS activity. As a result, less ROS are generated, and the aging process is delayed. However, this antiaging mechanism in stem cells was inhibited by Notch3‐ and FIH1‐mediated AIMP3 induction with hypoxia. The small RNA interference assays conducted under normoxia strongly support that AIMP3 is a key modulator in the autophagy‐associated antiaging pathway as well as mitochondrial metabolism

Here, we describe a novel mechanism regulating AIMP3 in stem cells in response to oxygen availability: in hypoxic conditions, HIF1α and Hey1 suppress AIMP3 expression and stem cell aging, whereas Notch3 show opposite effects (Figures 5 and 6). The direct transcriptional suppression of AIMP3 mediated by HIF1 is plausible upon our results and analysis: (a) The AIMP3 promoter contains an incomplete HRE sequence at −604 bp from a start codon, and a ChIP assay showed the existence of a HIF1α binding site (Figure 3b,d); (b) a putative binding site for a complex of aryl hydrocarbon receptor nuclear transporter (ARNT: HIF1β) and an aryl hydrocarbon receptor (AHR) exists at the −120 bp position, but the region was not detected by a HIF1α antibody, indicating that the precipitated fragment is specific for the HIF1α antibody against a HIF complex; and (c) both si‐HIF1α treatment and a HIF1α suppressor FIH1 induced AIMP3 expression. Although HIF1α‐Hey1 complex‐mediated regulation is still plausible, the failure to detect their interaction in our system suggests that their synergistic suppression of AIMP3 comes from different regulatory pathways in AIMP3 expression (Figure 4b). Contrary to the role of HIF1α and Hey1, Notch3 was first reported to enhance AIMP3 expression in this study (Figures 4 and 5). The Notch‐associated regulatory mechanisms in stem cells are strongly dependent on cellular contexts, resulting in a large spectrum of outcomes ranging from stem cell expansion and survival to differentiation, senescence, and cell death. Notch3 inhibits tumorigenesis by inducing p53‐p21‐associated cellular senescence of many human cells and suppresses the proliferation of placental trophoblast cells, whereas it enhances the tumor progression of human prostate cancers (Cui et al., 2013; Danza et al., 2013; Liu, Sato, Cerletti, & Wagers, 2010). Considering that the interaction of Notch3 and the AIMP3 promoter is undetectable, it is assumed that the Notch3‐mediated regulation is not direct. Interestingly, Raf kinase inhibitory protein (RKIP), an endogenous inhibitor of ERK, was recently reported to be negatively regulated by miR543, an AIMP3 suppressor (Du et al., 2017; Huttlin et al., 2017). Because RKIP binds to the Notch receptor and blocks its cleavage into the intracellular domain (NICD), inhibiting transcriptional activity, it is possible that miR543 modulates stem cell aging through RKIP‐associated Notch regulation and direct AIMP3 suppression.

In addition to AIMP3‐LMNA‐mediated cellular aging, we discovered that autophagy plays a key role in AIMP3‐associated cellular senescence: HIF1α‐Hey1‐mediated AIMP3 suppression induces autophagy and restricts mitochondrial respiration, which consequently inhibits stem cell aging (Figure 6). Contrary to the siAIMP3‐mediated induction of autophagy, the siAIMP3‐treated hpMSCs exhibited a reduction in mitochondrial respiration and p53 expression, resulting in reduced ROS levels and ROS‐induced damage (Figure 5a). Autophagy is an evolutionarily conserved cellular process that removes damaged macromolecules and organelles and modulates bioenergetic demands and survival under stress. In various somatic and stem cells, autophagy causes the bioenergetic shift from mitochondrial oxidation to glycolysis and promotes the transcriptional activation responsible for stemness, proliferation, and pluripotency (Guan et al., 2013). Recently, hematopoietic stem cells (HSCs) from conditionally autophagy‐deficient mice exhibited the accumulation of aged HSCs and accelerated blood aging: The affected HSCs displayed increased mitochondrial respiration, perturbed epigenetic status, and reduced stemness and regenerative potential. Interestingly, all old HSCs showing the activation of autophagy are healthy stem cells, implying that autophagy activation and cellular status are mutually influenced (Ho et al., 2017). Both of the previous reports and ours suggest that the fates of stem cells under hypoxia are determined by interactions among HIF1α‐associated hypoxia pathways, energy metabolism, including mitochondrial respiration, and autophagy‐involved cellular homeostasis: HIF1α induces pluripotency‐ and glycolysis‐related genes and represses mitochondrial biogenesis, which reduces cellular damage and enhances the potential for self‐renewal and pluripotency. Once cellular damage accumulates, stem cells activate autophagy to remove cellular detriments and maintain their stemness (Wanet, Arnould, Najimi, & Renard, 2015). The endogenous AD‐MSCs from AIMP3 TGs were less responsive to the autophagy‐inducing rapamycin; however, while autophagy in AD‐MSCs from WT mice was increased by approximately twofold by rapamycin, the cells from AIMP3 TGs did not exhibit rapamycin‐mediated autophagy induction (Figure 5b). Because only healthy HSCs are able to activate autophagy (Ho et al., 2017), AIMP3 overexpression may compromise stem cell functions and inhibit autophagy in AD‐MSCs even from 3‐month‐old mice. The mechanism of the AIMP3‐induced disturbance of autophagy is still under investigation, but our results clearly show that AIMP3 is a crucial regulator of autophagy‐associated antiaging mechanisms in stem cells. Whether rapamycin is able to relieve the progeroid deficits manifested in AIMP3 TG mice is an interesting question because rapamycin‐mediated autophagy induction rescued premature aging of a rodent model of progeria, including Hutchinson–Gilford progeria syndrome (HGPS), through the degradation of mutated lamin A protein (Blagosklonny, 2011). Our results suggest that rapamycin may not be efficient without the modulation of AIMP3 levels and that the novel HIF1α‐Notch3‐mediated AIMP3 regulation is a key pathway for developing antiaging interventions.

In conclusion, we present a novel regulatory mechanism of AIMP3 under hypoxia and an AIMP3‐associated autophagy pathway in stem cell aging. Because multiple molecules, including HIF1α, Notch3, and autophagic molecules, are associated with AIMP3‐implicated stem cell aging, the results here provide us with several possible targets for developing interventions against aging as well as expanding our knowledge about aging processes.

## 4 EXPERIMENTAL PROCEDURES

### 4.1 Isolation and culture of hpMSCs

Human full‐term placentas (≥37 gestational weeks) were obtained by Caesarean section as previously described (Kim et al., 2013). All donors provided written, informed consent prior to donation. The collection of the samples and their use for research purposes were approved by the Institutional Review Board (IRB) of CHA General Hospital, Seoul, Korea.

### 4.2 Growth curve and population doubling time and flow cytometry analysis

Described in detail in the Supporting Information.

### 4.3 Quantitative reverse transcription PCR and immunoblot analysis

Described in detail in the Supporting Information.

### 4.4 Adipogenic, osteogenic, and chondrogenic differentiation of hpMSCs

Described in detail in the Supporting Information.

### 4.5 Recombinant plasmid and siRNA transfection

Described in detail in the Supporting Information. The full‐length AIMP3‐expressing plasmid (pBiFC‐VN173‐AIMP3) was generously provided by Dr. Sunghoon Kim at the Seoul National University. The siRNA sequences are provided in the Supporting Information.

### 4.6 Chromatin immunoprecipitation assay

Described in detail in the Supporting Information.

### 4.7 AIMP3‐overexpressing transgenic mice

AIMP3 transgenic mice (AIMP3 TG) were generously gifted from Dr. Sunghoon Kim at the Seoul National University. All experimental animals were housed in specific pathogen‐free conditions (CHA Laboratory Animal Research Center) and handled in accordance with an animal protocol approved by the CHA University Institutional Animal Care and Use Committee (IACUC number: 180091). Other procedures are described in detail in the Supporting Information.

### 4.8 Isolation and culture of primary adipose‐derived mesenchymal stem cells from AIMP3 TG mice

Three‐ and 10‐month‐old AIMP3 TG and littermate female control mice were sacrificed for the isolation of adipose‐derived mesenchymal stem cells (AD‐MSCs). For autophagy induction, the AD‐MSCs from 3‐month‐old mice were treated with rapamycin (300 nmol; Sigma) for 24 hr, and the cell lysates were collected for Western blot analysis. Other procedures are described in detail in the Supporting Information.

### 4.9 Western blot analysis

Described in detail in the Supporting Information.

### 4.10 mRNA sequence analysis (R‐seq) and functional annotation of hpMSCs cultured under normoxia and hypoxia

The messenger RNAs were isolated from hpMSCs at p10 cultured either under normoxia or hypoxia, and differentially expressed genes (DEG) were analyzed. After identifying DEG between the two conditions, the DEGs were sorted based on 305 human aging genes from the “Aging gene” database: genes of +1.5 log2 or −1.5 log2 fold change under hypoxia compared to those under normoxia were designated as upregulated or downregulated genes, respectively. The analysis is described in detail in the Supporting Information.

### 4.11 Statistics

Statistical analyses were conducted with a CHA University mainframe computer using the Statistical Package for the Social Sciences (IBM SPSS Statistics, version 22.0; IBM Korea, Inc., Seoul, Korea) and described in detail in the Supporting Information.

## CONFLICT OF INTEREST

The authors declare that no conflicts of interest exist.

## AUTHOR CONTRIBUTIONS

C.K. and J.‐M.P. designed and performed the experiments, analyzed the results, and wrote the manuscript. Y.S. cared for the animals, prepared the tissues, and performed the experiments with primary tissues. S.K. and J.M. supervised all experiments and the writing of the manuscript.
