# Supplementary figures and images for: HIF1α‐mediated AIMP3 suppression delays stem cell aging via the induction of autophagy
Source: Aging Cell. 2019 Jan 31;18(2):e12909. doi: 10.1111/acel.12909 (PMC6413650; doi:10.1111/acel.12909)

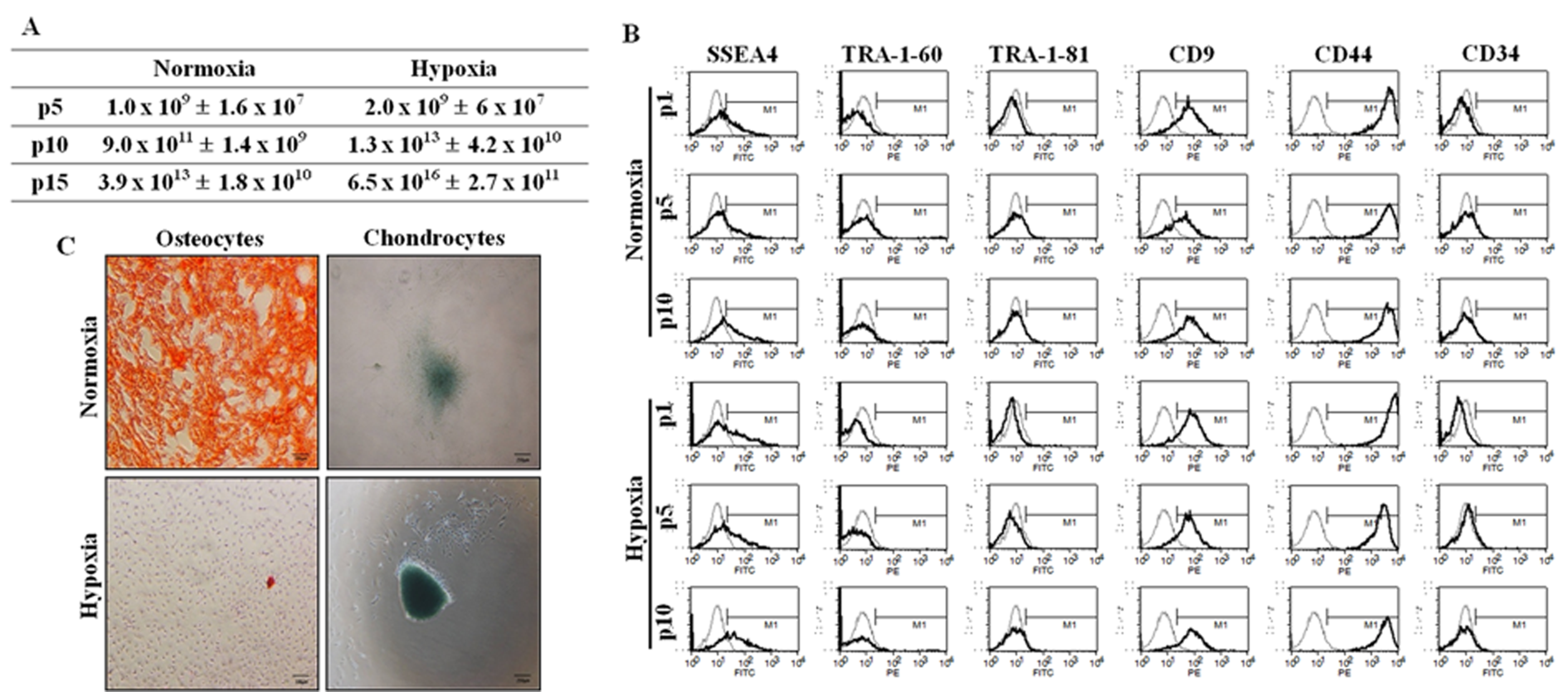

Supplement: Supplementary file 1 [file ACEL-18-e12909-s001.zip › Suppl Fig. 1.tif]

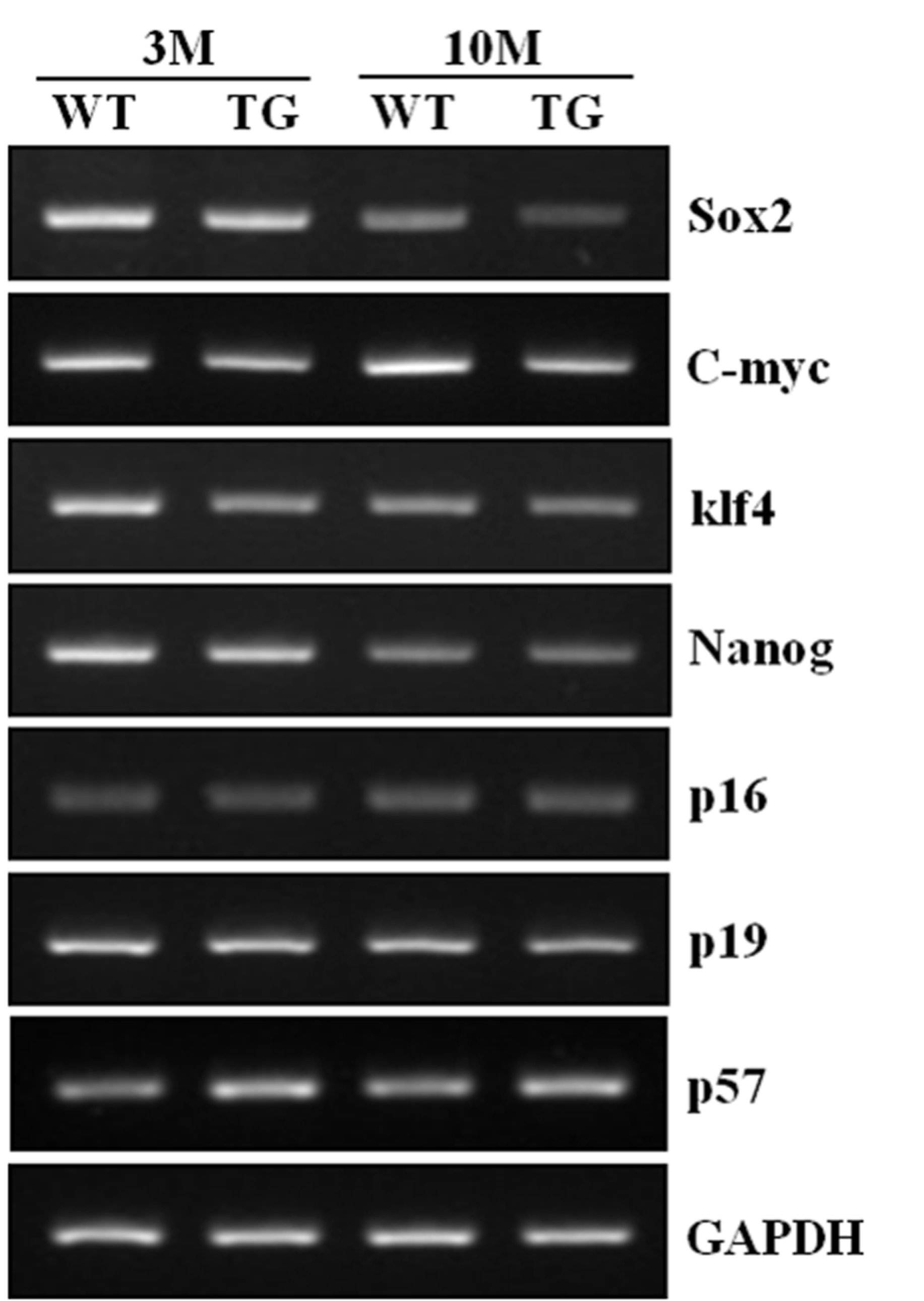

Supplement: Supplementary file 1 [file ACEL-18-e12909-s001.zip › Suppl Fig. 2.tif]

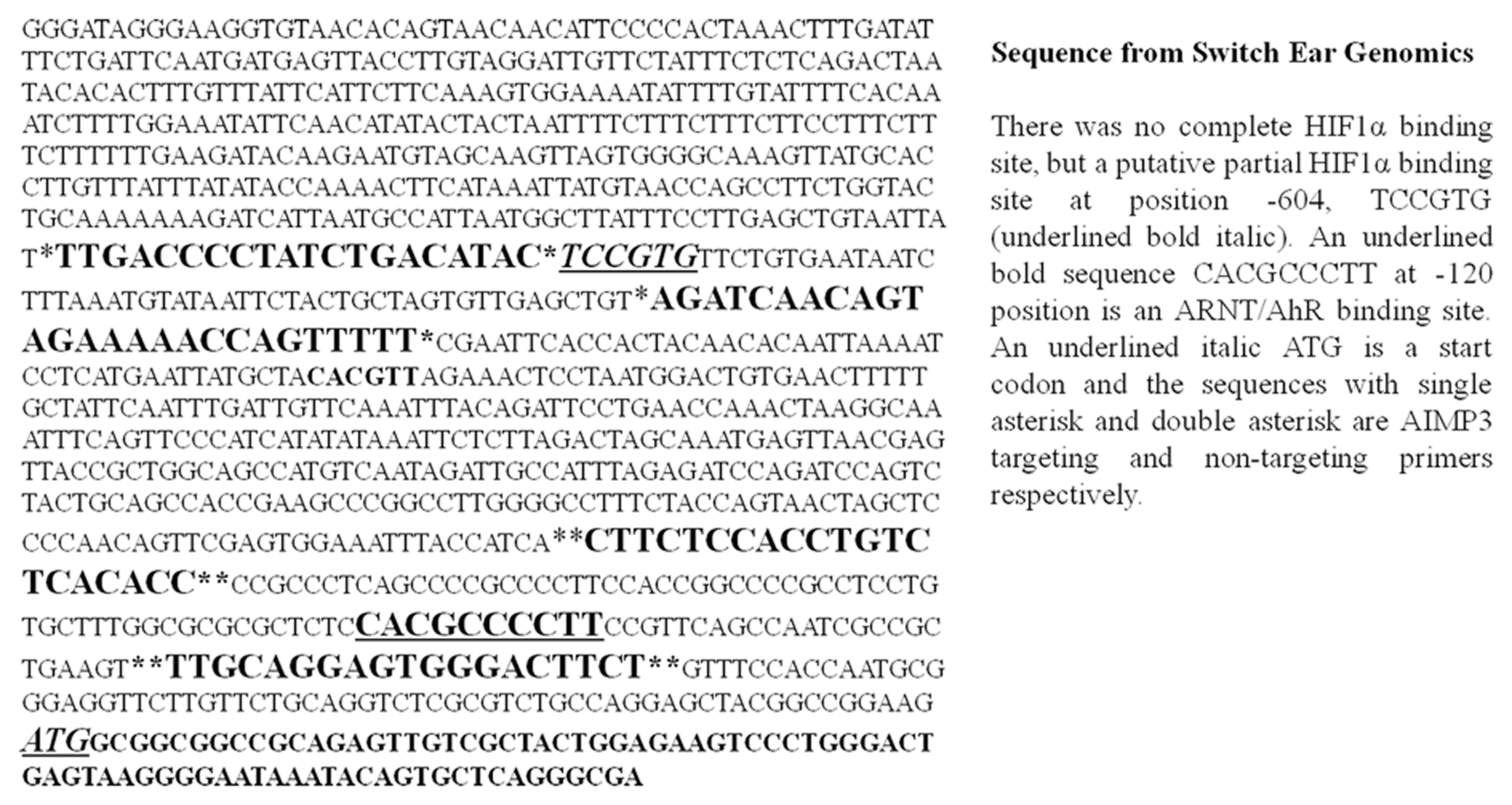

Supplement: Supplementary file 1 [file ACEL-18-e12909-s001.zip › Suppl Fig. 3.tif]
