# Supplementary material for: HIF1α‐mediated AIMP3 suppression delays stem cell aging via the induction of autophagy
Source: Aging Cell. 2019 Jan 31;18(2):e12909. doi: 10.1111/acel.12909 (PMC6413650; doi:10.1111/acel.12909)
Supplement: Supplementary file 1 [file ACEL-18-e12909-s001.zip › Aging Cell_Moon et al._Supplementary Information_FINAL.doc]

***Supporting Information***

***Experimental Procedures***

***Isolation and culture of hpMSCs:*** Human full-term placentas (≥37 gestational weeks) were obtained by Caesarean section as previously described (Kim et al., 2013). All donors provided written, informed consent prior to donation. The collection of the samples and their use for research purposes were approved by the Institutional Review Board (IRB) of CHA General Hospital, Seoul, Korea.

***Growth curve and population doubling time (PDL):*** For the accumulation growth curves of hpMSCs, the hpMSCs from passages 1-15 were seeded at 5x10^5^ cells/75 T density with culture medium. The hpMSCs were harvested and counted at 3 days. The number of accumulated cells was calculated with the formula for the population doubling time (PDL). Td = log (2) / log (1 + r) (Td = doubling time; r = a constant growth rate)

***Flow cytometry analysis:*** hpMSCs (1x10^5^ of each sample) were resuspended in 75 ml phosphate-buffered saline (PBS; pH 7.4) and incubated with 0.1 mg of the primary antibodies against human SSEA-4, TRA-1-60, TRA-1-81, CD9, CD34, and CD44 for 60 min at 37°C. For the negative controls, the cells were incubated without primary antibody. Cells were washed with 1 ml PBS and 1% heat-inactivated fetal bovine serum. After resuspension in 300 ml PBS, samples were analyzed by flow cytometry using FACScan (BD, Franklin Lakes, NJ, http://www.bd.com). The sample flow rate during analysis did not exceed 300–400 cells per second.

***mRNA sequence analysis (R-seq) and functional annotation with hpMSCs cultured under normoxia and hypoxia:*** The messenger RNAs were isolated from hpMSCs at p10 cultured either under normoxia or hypoxia, and the raw read data generated from Ion Proton sequencer were filtered to exclude data of poor quality. Read alignment was performed using STAR v. 2.3.0 aligner (*Bioinformatics*) on the UNIX platform with default parameters, producing alignments in the SAM format that were converted to a BAM file and sorted by SAM tools (*Bioinformatics*) for subsequent analysis. Genome and transcript references were obtained from the Ensemble database. The unmapped reads from the first alignment step were realigned with bowtie2 (ultrafast and memory-efficient alignment of short DNA sequences to the human genome, *Genome Biology*) to increase the mapping rate. HTSeq-count program (HTSeq—a Python framework to work with high-throughput sequencing data, *Bioinformatics*) was used to obtain raw gene-level read counts with intersection-nonempty mode. The gene-level read counts were used as the input for DESeq (differential expression analysis for sequence count data, *Genome Biology*) to normalize the counts and analyze differentially expressed genes (DEG). The difference in gene expression was considered to be statistically significant at p-values of less than 0.001. Gene ontology (GO) annotation was performed to assign putative biological function to gene products using DAVID .

***Quantitative reverse transcription PCR:*** Total RNA was isolated from cells using TRIzol (Invitrogen). cDNA was synthesized from 1 μg of total RNA using Superscript II reverse transcriptase with Oligo-d(T) 12-18 primer as recommended in the manufacturer’s protocol. RT-PCR was conducted using a pre-mix kit (Bioneer) according to the vendor’s manual. The PCR products were subjected to 2% agarose gel electrophoresis with RedGel (Biotium, Inc.) and visualized on a UV transilluminator.

***Immunoblot analysis:*** Cells were washed with cold PBS and lysed in a lysis buffer containing a protease inhibitor cocktail (Pierce). Whole cell lysates were normalized using the Bradford reagent (Bio-Rad), and 20~50 μg of the lysate was subjected to 10% SDS-PAGE and transferred to a PVDF membrane (Millipore). Membranes were blocked in TBST (50 mM Tris-HCl, pH 7.6, 150 mM NaCl, 0.05% Tween-20) containing 10% skim milk (Becton Dickinson) and then probed with each antibody. Immunoreactions were conducted using the ECL Western Blotting system (Millipore) and visualized by Ez-Capture MG (ATTO).

***Recombinant plasmid and si-RNA transfection:*** An AIMP3-expressing plasmid (pBiFC-VN173-AIMP3) was prepared by cloning the full-length human AIMP3 into pBiFC-VN173 at the EcoRI/XhoI site. The plasmid was provided by Dr. Sunghoon Kim at the Seoul National University. The HIF1α-expressing plasmid (pBABE-HIF1a) was prepared by cloning the full-length human HIF1α in pBABE at the BamHI/Not1 site. The plasmid was purchased from Addgene. The siRNAs targeting human AIMP2, AIMP3, HIF1α and Hey1 were purchased from Bioneer with sequences of AIMP2. The cell transfection with siRNA and recombinant plasmids was performed using a Lipofectamine 2000 (Invitrogen) according to the manufacturer’s protocol. Four hours after transfection, the media were completely replaced and incubated further. The siRNA sequences are provided in the supplemental information.

***Chromatin immunoprecipitation (ChIP) assay:*** ChIP assays were performed according to the manufacturer’s protocol (EZ-ChIP, Millipore). Formaldehyde-cross-linked chromatin was sonicated to generate a mean fragment size and divided into aliquots. DNA from one aliquot was isolated following reverse cross-linking and represented the total input DNA available for immunoprecipitation. The other aliquots were precleared and incubated with anti-HIF1α and anti-normal mouse IgG and precipitated with protein G agarose. After washing, the chromatin was eluted, and cross-links were reversed. Purified DNA from the immunoprecipitates, as well as from total input DNA, was analyzed by PCR.

***Differentiation potential analysis of hpMSCs***

***Adipogenic differentiation:*** The cells at passages 4-5 were seeded at a density of 1.5×10^4^ cells/cm^2^ in 6-well culture dishes and cultured in alpha-MEM (Gibco) with 10% FBS (Gibco), 25 ng/ml FGF4 (R&D Systems), and 1 μg/ml heparin (Sigma) until they reached 100% confluence. The cells were then subjected to induction/maintenance by sequentially using adipogenic induction medium (StemPro Adipocyte Differentiation Medium, Gibco) for 7 days. After differentiation, the cells were fixed with 10% formalin, washed, and stained with 2% (wt/vol) Oil Red O reagent (Sigma) for 5 min at room temperature to examine the generation of oil droplets in the cytoplasm.

***Osteogenic differentiation:*** The cells at passages 4-5 were seeded at a density of 3×10^3^ cells/cm^2^, cultured in alpha-MEM (Gibco) with 10% FBS (Gibco), 25 ng/ml FGF4 (R&D Systems), and 1 μg/ml heparin (Sigma) until they reached 70-80% confluence, and then fed twice per week for 2.5 weeks with osteogenic induction medium (StemPro Osteogenic Differentiation Medium, Gibco). The osteogenic differentiation was detected by fixing the cells with 10% formalin for 15 min at room temperature and then staining them with the von Kossa stain, which demonstrates the deposition of a hydroxyapatite matrix.

***Chondrogenic differentiation:*** To induce chondrogenic differentiation, cells at passages 4-5 were initially seeded (3×10^3^ cells/cm^2^) and then transferred into 15 ml polypropylene tubes, centrifuged at 1000 rpm for 5 min to form a pelleted micromass at the bottom of each tube, and finally treated with chondrogenic medium (StemPro Chondrogenic Differentiation Medium, Gibco). Medium changes were performed twice weekly, and chondrogenesis was assessed weekly. After 2~4 weeks in culture, the cells were washed twice with PBS, fixed in 4% PFA, and stained with Alcian Blue.

***Isolation of primary adipose-derived mesenchymal stem cells (AD-MSCs) and cultivation:*** Three- and 10-month-old AIMP3 TG and littermate female control mice were sacrificed for isolation of mAD-MSCs. The adipose tissues collected from the mammary glands of females and from the testes and epididymis of males were washed in PBS and digested at 37°C for 60 min in DMEM/F12 (Gibco) containing 1 mg/ml collagenase type I (Gibco) with constant shaking. Cells were filtered through a 100 μm cell strainer (Falcon) and centrifuged for 5 min at 1500 rpm. Red blood cells (RBCs) in the stromal vascular fraction (SVF) were lysed in RBC lysis buffer. Cells were centrifuged for 5 min at 1500 rpm, and the cell pellet was resuspended in DMEM/F12 supplemented with 10% FBS (Gibco) and 100 µg/ml antibiotics (Gibco). Cells were filtered through a 40 μm cell strainer (Falcon) and centrifuged for 5 min at 1200 rpm. The cell pellet was resuspended in growth media and cultured as a monolayer at 37°C under hypoxic conditions (92% N_2_, 5% CO_2_, and 3% O_2_).

***Statistics:*** PDL and the number of Ki-positive cells were analyzed using either a t-test or a 2-way variance analysis followed by Fisher’s least significant difference post hoc tests. All other measures were analyzed using a mixed model analysis of variance procedure to account for the random effect of the mice. Data are presented as the mean ± standard error of the mean, and a p value of < 0.05 was considered significant.

***Supplemental Figures Legends***

**Supplemental Figure 1. The hpMSCs under hypoxia maintained the ability to proliferate and exhibited induced chondrocyte genesis**

A. Differences in the accumulated numbers of cells between normoxia and hypoxia were observed from p5, and the number of hpMSCs under hypoxia at p15 was 2000 times higher than that under normoxia.
B. Flow cytometry analysis of hpMSCs cultured either under normoxia or hypoxia. The hpMSCs in both conditions displayed an absence of embryonic stem cell (ESC) markers and high expression of MSC markers up to p10
C. The hpMSCs cultured under either normoxia or hypoxia were driven to differentiate into osteoblasts or chondrocytes. Osteoblast differentiation was enhanced and chondrocyte differentiation was suppressed in normoxia. The hpMSCs at p4 were treated with proper conditions. The scale bars represent 100 μm for osteoblasts, 250 μm for chondrocytes, and 50 μm for adipocytes.

**Supplemental Figure 2. The stem cells derived from AIMP3 TG mice exhibited compromised stem cell properties**

In the AD-MSCs from 3M and 10M AIMP3 TG mice, four stem cell-specific transcription factors were suppressed, and the senescence inducer p57 was induced compared to the age-matched controls.

**Supplemental Figure 3. The AIMP3 promoter contains a putative HIF1α binding site**

The AIMP3 promoter region (-1002 from the start codon) was acquired from Switch Ear Genomics and analyzed to understand the underlying mechanism of HIF1α-mediated AIMP3 regulation. There was no complete HIF1α binding site but a putative partial HIF1α binding site at position -604, ***TCCGTG*** (underlined bold italic). The underlined bold sequence of CACGCCCTT at the -120 position is an ARNT/AhR binding site. An underlined italic ATG is the start codon, and the sequences with single asterisks and double asterisks are AIMP3 targeted and nontargeted primers, respectively.

**Supplemental Table 1. List of aging-related genes induced in hpMSCs under low oxygen pressure**

Huang da, W., Sherman, B. T., & Lempicki, R. A. (2009). Systematic and integrative analysis of large gene lists using DAVID bioinformatics resources*. Nat Protoc,* 4(1), 44-57. doi:10.1038/nprot.2008.211
