# Supplementary material for: HIF1α‐mediated AIMP3 suppression delays stem cell aging via the induction of autophagy
Source: Aging Cell. 2019 Jan 31;18(2):e12909. doi: 10.1111/acel.12909 (PMC6413650; doi:10.1111/acel.12909)
Supplement: Supplementary file 1 [file ACEL-18-e12909-s001.zip › Suppl Table 1.doc]

| **GO enrichment of upregulated aging-associated genes** | **Genes** |
| --- | --- |
| **Positive regulation of transcription,**  **DNA-templated** | ***INSR, PDGFB, FOS, RET, TCF3, BLM, EGFR1, BRCA2, TGFB1, FOXM1, CCNA2, HMGB2, TFAP2A, PPARGC1A, FGF23, PPARG, TP72*** |
| **Aging** | ***KL, NFKB2, JUND, FOS, TGFB1, EEF2, RGN, FOXO4, UCP3, PPARGC1, IGFBP2, CNR1*** |
| **Response to estradiol** | ***PDGFRB, TGFB1, EEF2, PTGS2, LEP, SOCS2, NCOR2, IGFBP2*** |
| **Response to drug** | ***JUND, FOS, RET, TGFB1, HMGB2, PTGS2, PPARGC1A, PPARG, IGFBP2, TP73*** |
| **MAPK cascade** | ***KL, IRS1, IRS2, PDGFB, IL2RG, PDGFRB, RET, TGFB1, FGF23*** |
| **Positive regulation of transcription**  **from RNA polymerase II promoter** | ***NFKB2, JUND, FOS, TCF3, EGR1, TOP2A, CEBPB, TGFB1, FOXM1, HMGB2, FOXO4, TFAP2A, PPARGC1A, PPARG, TP73*** |
| **Positive regulation of cell**  **proliferation** | ***IGF2, IRS1, IRS2, INSR, PDGFB, PDGFRB, TGFB1, FOXM1, IL7, LEP,***  ***CTF1*** |
| **Negative regulation of transcription from RNA polymerase II promoter** | ***NFKB2, JUND, TCF3, EGR1, TGFB1, FOXM1, TFAP2A, LEP, HIC1,***  ***PPARG, NCOR2, TP73, TRPV1*** |
| **Regulation of phosphatidylinositol**  **3-kinase signaling** | ***KL, IRS1, IRS2, PDGFB, PDGFRB, FGF23*** |
| **Insulin receptor signaling pathway** | **KL, IGF2, IRS1, IRS2, INSR, FOXO4** |
